# Masked Triple-Negative Polycythemia Vera Initially Presenting as Iron Deficiency and Pernicious Anemia

**DOI:** 10.7759/cureus.116982

**Published:** 2026-09-26

**Authors:** Oren G Nedjar, Ismael Palacio, David Israilov, Zaneh Kahook, Marvi Alais Surksha, Alex Wu, Ambar M Rodriguez, Lisandra Inastrilla, George Michel

**Affiliations:** 1 Internal Medicine, Nova Southeastern University Dr. Kiran C. Patel College of Osteopathic Medicine, Fort Lauderdale, USA; 2 Internal Medicine, Larkin Community Hospital, Miami, USA; 3 Rheumatology, Nova Southeastern University Dr. Kiran C. Patel College of Osteopathic Medicine, Fort Lauderdale, USA; 4 Neurology, Nova Southeastern University Dr. Kiran C. Patel College of Osteopathic Medicine, Fort Lauderdale, USA; 5 Medicine, Larkin Community Hospital, Miami, USA; 6 Hematology and Oncology, Larkin Community Hospital, Miami, USA

**Keywords:** autoimmune gastritis, iron deficiency anemia, jak2-unmutated, masked polycythemia vera, myeloproliferative neoplasm, pernicious anemia, polycythemia vera

## Abstract

Polycythemia vera (PV) is a myeloproliferative neoplasm characterized by erythrocytosis, typically driven by *JAK2* mutations. Diagnosis can be challenging when concurrent conditions mask the underlying erythrocytosis. Iron deficiency is the most common masking factor; however, to the authors’ knowledge, this is the first reported case in which the combination of iron deficiency and pernicious anemia created a dual masking mechanism. A 68-year-old Hispanic female patient with a history of chronic anemia presented with severe symptomatic anemia requiring transfusion (hemoglobin 5.2 g/dL). Evaluation revealed iron deficiency anemia and pernicious anemia secondary to autoimmune gastritis, confirmed by positive intrinsic factor and anti-parietal cell antibodies. Following intravenous iron and intramuscular vitamin B12 repletion, hemoglobin normalized. However, the patient subsequently developed progressive erythrocytosis, with hemoglobin rising to 19.8 g/dL and hematocrit to 58.7%. Evaluation revealed suppressed erythropoietin (2.1 mIU/mL), negative molecular testing for *JAK2*, *CALR*, and *MPL* mutations, and exclusion of secondary causes. Repeat bone marrow biopsy demonstrated marked hypercellularity with trilineage panmyelosis and clusters of enlarged pleomorphic megakaryocytes with hyperlobulated nuclei, establishing the diagnosis of *JAK2*-unmutated PV. This case illustrates a rare presentation of masked PV in which concurrent iron deficiency and pernicious anemia from autoimmune gastritis simultaneously obscured the underlying myeloproliferative process. The dramatic hemoglobin swing from 5.2 g/dL to 19.8 g/dL, observed during clinical follow-up after correction of the iron and vitamin B12 deficiencies, served as the key diagnostic clue. Clinicians should maintain vigilance for underlying myeloproliferative neoplasms when erythrocytosis develops following correction of nutritional deficiencies, even in the absence of canonical driver mutations. In *JAK2*-unmutated cases, bone marrow morphology becomes the cornerstone of diagnosis.

## Introduction

Polycythemia vera (PV) is a chronic myeloproliferative neoplasm characterized by clonal proliferation of myeloid cells, leading to increased red blood cell mass, often accompanied by leukocytosis, thrombocytosis, and splenomegaly [1]. The disease is driven primarily by gain-of-function mutations in *JAK2*, most commonly the V617F mutation in exon 14, which is present in more than 95% of affected individuals and leads to constitutive activation of the JAK-STAT signaling pathway [1,2]. The annual incidence is estimated at 0.5 to 4.0 per 100,000 individuals in the United States, with a median age at diagnosis of 65 years [1].

Despite well-defined diagnostic criteria, PV can be challenging to diagnose when erythrocytosis is obscured by concurrent conditions, a phenomenon termed "masked PV." Iron deficiency is the most common masking factor and is present in most patients at diagnosis or during the disease course [3-5]. Dysregulated iron metabolism in PV is characterized by insufficiently suppressed hepcidin levels relative to the degree of iron deficiency, which may be influenced by inflammatory cytokines, creating a disconnect between red cell proliferation and hemoglobin synthesis [3,6]. In these patients, hemoglobin may appear normal or low despite an increased red blood cell mass, as iron restriction reduces hemoglobin content per cell while the total number of red cells remains elevated [4]. Additionally, PV frequently causes plasma volume expansion, which further masks the true erythrocytosis by diluting the red cell concentration; in many patients, especially women, the hematocrit appears normal despite a significantly elevated red cell mass [4]. Masked PV occurs in approximately 15% to 35% of cases depending on classification criteria and is associated with significantly worse overall survival compared with overt PV [5,7]. Once iron stores are replenished, the underlying erythrocytosis becomes clinically apparent as hemoglobin and hematocrit levels increase [3].

Autoimmune gastritis represents a unique mechanism by which both iron deficiency and vitamin B12 deficiency can coexist, creating a dual masking effect. In autoimmune gastritis, pathologic Th1 CD4 T cells directed against gastric H+/K+ ATPase lead to the destruction of parietal cells, resulting in achlorhydria and loss of intrinsic factor production [8,9]. Loss of gastric acid impairs iron absorption, as gastric acid is required to reduce dietary ferric iron to the absorbable ferrous form, while loss of intrinsic factor leads to vitamin B12 malabsorption and pernicious anemia [8,10]. Importantly, iron deficiency typically develops first and may precede vitamin B12 deficiency by years to decades, explaining the progression from microcytic to macrocytic anemia observed in some patients [8]. The simultaneous presence of iron deficiency causing microcytosis and vitamin B12 deficiency causing macrocytosis may result in a normal mean corpuscular volume, further complicating diagnosis [11].

Major criteria for the diagnosis of PV include elevated hemoglobin or hematocrit (greater than 16.5 g/dL in men or greater than 16.0 g/dL in women, or hematocrit greater than 49% in men or greater than 48% in women), bone marrow hypercellularity with trilineage proliferation and pleomorphic megakaryocytes, and *JAK2* V617F or exon 12 mutation [1,12]. A minor criterion is subnormal serum erythropoietin. Diagnosis requires either all three major criteria or the first two major criteria plus the minor criterion [1,12]. Bone marrow biopsy remains essential in challenging cases, especially masked PV, as trilineage proliferation with pleomorphic megakaryocytes is highly specific and helps distinguish PV from secondary erythrocytosis [5]. In the small minority of patients (approximately 2%) who lack *JAK2*, *CALR*, and *MPL* mutations, diagnosis relies primarily on clinical findings, subnormal erythropoietin, and characteristic bone marrow morphology after exclusion of secondary and hereditary causes of erythrocytosis [1].

This case report describes an exceptionally rare and diagnostically complex presentation of PV characterized by three overlapping features that together created a significant diagnostic challenge. First, the patient demonstrated *JAK2*-unmutated PV, defined by the absence of *JAK2* V617F, *JAK2* exon 12, *CALR*, and *MPL* mutations. Second, the patient had concurrent iron deficiency anemia secondary to autoimmune gastritis, which masked the underlying erythrocytosis. Third, the patient also had pernicious anemia from the same autoimmune gastritis, creating a dual masking mechanism in which both iron deficiency and ineffective erythropoiesis from vitamin B12 deficiency obscured the underlying myeloproliferative process. This case illustrates the importance of maintaining clinical suspicion for an underlying myeloproliferative neoplasm when erythrocytosis develops following correction of nutritional deficiencies, even in the absence of identifiable driver mutations.

## Case presentation

We present the case of a 68-year-old Hispanic female patient with a past medical history of chronic anemia, gastritis, type 2 diabetes mellitus, hypertension, anxiety, and depression. Her surgical history was notable for left knee replacement. Her family history was significant for pancreatic cancer in her mother, who died in her 60s. The patient's home medications included aspirin 81 mg once daily.

The patient presented to the Hematology Oncology clinic in May 2020 for evaluation of severe symptomatic anemia requiring transfusion. She reported a longstanding history of anemia dating back to early adulthood, with multiple prior episodes requiring blood transfusions. Most recently, she had received packed red blood cell transfusions for hemoglobin levels of 6.1 g/dL in September 2019 and 5.2 g/dL in May 2020. At presentation, she endorsed fatigue, pica, and intermittent dizziness, without overt bleeding or constitutional symptoms. Previous attempts at oral iron supplementation had been discontinued due to intolerance, manifesting as gastritis and diarrhea.

Initial laboratory evaluation demonstrated low serum iron and ferritin with elevated transferrin and total iron-binding capacity, consistent with iron deficiency anemia (Table 1). Due to intolerance to oral iron, intravenous iron supplementation was initiated. Following six doses by late June 2020, the patient demonstrated marked clinical and hematologic improvement, with resolution of pica and significant reduction in fatigue.

**Table 1:** Iron studies at diagnosis and during repletion TIBC, total iron-binding capacity

Date	Serum Iron	TIBC	Ferritin	Interpretation
April 2021	124	282	800	Iron repletion
Mid-September 2020	86	383	54.4	Improving iron stores
Late September 2020	-	-	192.4	Iron repletion
May 2020	19	530	17	Severe iron deficiency
Reference range	60–170 µg/dL	250–450 µg/dL	15–150 ng/mL (female)	-

Given the severity of anemia disproportionate to iron deficiency alone, further evaluation was pursued, revealing concomitant vitamin B12 deficiency. Additional testing confirmed pernicious anemia with positive intrinsic factor and anti-parietal cell antibodies (Table 2). The patient was started on intramuscular cyanocobalamin (1,000 mcg monthly) in late October 2020, with subsequent clinical improvement.

**Table 2:** Pernicious anemia diagnosis workup in late October 2020 MMA, methylmalonic acid; TIBC, total iron-binding capacity

Test	Result	Interpretation	Reference range
Vitamin B12 (pg/mL)	<108	Severe deficiency	200–900 pg/mL
Intrinsic factor antibody	Positive	Supports pernicious anemia	-
MMA (nmol/L)	1419	Elevated	87–318 nmol/L
Ferritin (ng/mL)	110	Normal	15–150 ng/mL (female)
TIBC (µg/dL)	864	Elevated	250–450 µg/dL

Following iron and vitamin B12 repletion, the patient demonstrated progressive hematologic recovery, with normalization of hemoglobin and resolution of symptoms (Table 3). She remained clinically well on maintenance therapy through early 2023.

**Table 3:** Complete blood count trend WBC, white blood cell count; MCV, mean corpuscular volume; ANC, absolute neutrophil count; ALC, absolute lymphocyte count

Date	WBC (×10³/µL)	Hemoglobin (g/dL)	Hematocrit (%)	MCV (fL)	Platelets (×10³/µL)	ANC (×10³/µL)	ALC (×10³/µL)
March 2026	9.5	19.8	58.7	87	550	6.4	2.1
Late January 2026	9.6	18.7	56.5	87	462	7.1	1.6
Early January 2026	12.1	19.3	58.1	89	458	9.6	1.6
September 2025	11.4	18.4	54.7	-	402	8.4	2
July 2025	9	16.5	50.8	-	304	6.7	1.6
January 2025	10	17	52.4	-	269	6.9	2.4
December 2024	10.5	17.3	53.5	-	221	6.9	2
November 2024	11.5	18.3	56.6	-	217	8.4	2.2
Late May 2024	9.6	17.1	51.3	-	213	-	-
Early May 2024	13.5	18.2	53.7	-	256	10.3	-
December 2023	10.8	16.4	49.2	87	199	-	-
Early October 2023	10.1	18	54.8	87	210	6.7	2.4
April 2021	9.3	15.7	46.2	89	215	-	-
January 2021	9.39	15.8	47.1	89.4	239	5.73	2.1
Mid-October 2020	9.75	14.6	44.2	87.5	223	4.98	-
Late September 2020	9.45	14.9	45.9	86.1	249	5.26	-
Mid-September 2020	9.97	12.6	37.8	85	229	-	-
June 2020	10	10.7	32.1	79	306	-	-
May 2020	9.87	7.5	22.5	67.5	386	-	-
Reference range	4.0–10.5	12.0–16.0	36–46	80–100	150–400	1.5–8.0	1.0–4.0

Unexpectedly, following successful correction of her combined deficiency state, the patient developed progressive erythrocytosis, a finding that fundamentally shifted the diagnostic trajectory toward a primary myeloproliferative process. Hemoglobin rose from a previously normalized baseline to 17.1 g/dL and 18.2 g/dL in May 2024, and further increased to 18.3 g/dL in November 2024 (Table 3). By March 2026, hemoglobin had reached 19.8 g/dL with hematocrit of 58.7%. This paradoxical rise in hemoglobin following correction of prior anemia raised concern for an underlying primary hematologic process [1,5].

Evaluation for secondary causes of erythrocytosis was pursued. Polysomnography demonstrated primary snoring without evidence of obstructive sleep apnea. Renal ultrasound revealed no masses. Comprehensive metabolic evaluation remained largely unremarkable (Table 4).

**Table 4:** Comprehensive metabolic panel trend Abbreviations: BUN, blood urea nitrogen; GFR, estimated glomerular filtration rate; Na, sodium; K, potassium; Ca, calcium; Alk Phos, alkaline phosphatase; AST, aspartate aminotransferase; ALT, alanine aminotransferase.

Date	BUN (mg/dL)	Creatinine (mg/dL)	GFR (mL/min/1.73 m²)	Na (mmol/L)	K (mmol/L)	Ca (mg/dL)	Albumin (g/dL)	Alk Phos (U/L)	AST (U/L)	ALT (U/L)
March 2026	22	1.11	53	141	4.7	10.4	4.4	145	17	15
Late January 2026	25	0.97	63	140	5.3	10.5	4.4	133	19	19
Early January 2026	24	0.94	65	141	4.5	10.6	4.8	149	22	27
September 2025	28	1.19	49	141	4.8	10.7	4.7	126	18	20
September 2024	32	1.14	52	-	-	10.5	4.6	123	15	22
January 2024	24	1.09	55	-	-	10.6	4.5	138	18	23
Early October 2023	18	1.02	60	-	-	10.3	4.6	99	24	43
May 2020	27	1.03	57	-	-	-	-	-	-	-
Reference range	7–25 mg/dL	0.6–1.2 mg/dL	≥60 mL/min/1.73 m²	135–145 mmol/L	3.5–5.1 mmol/L	8.6–10.2 mg/dL	3.5–5.0 g/dL	35–104 U/L	10–40 U/L	7–56 U/L

Laboratory studies demonstrated a suppressed erythropoietin level of 2.1 mIU/mL, suggesting a primary erythrocytosis rather than secondary causes, which typically present with elevated erythropoietin levels [1]. Molecular testing for myeloproliferative neoplasm driver mutations (including *JAK2* V617F, *JAK2* exon 12, *CALR*, and *MPL*) was negative, raising the possibility of *JAK2*-unmutated PV (Table 5) [1].

**Table 5:** Molecular evaluation of erythrocytosis, hematologic, and endocrine studies EPO, erythropoietin; TSH, thyroid-stimulating hormone; PTH, parathyroid hormone; CALR, calreticulin; MPL, myeloproliferative leukemia virus oncogene; NGS, next-generation sequencing; LDH, lactate dehydrogenase

Date	Test	Result	Interpretation	Reference range
March 2026	Erythropoietin	2.1 (low)	Suppressed EPO level supporting primary erythrocytosis such as polycythemia vera	4.0–27.0 mIU/mL
Late January 2026	Vitamin B12	313	Within normal range following replacement therapy	200–900 pg/mL
Late January 2026	Folate	7	Within normal range	>4 ng/mL
August 2025	TSH	2.54	Normal thyroid function	0.4–4.5 mIU/L
August 2025	Free T4	1.2	Normal thyroid function	0.8–1.8 ng/dL
August 2025	Vitamin D	31	Borderline sufficient level	30–100 ng/mL
August 2025	PTH	45	Within normal range	15–65 pg/mL
July 2025	Erythropoietin	5.1 (low-normal)	Inappropriately low-normal in the setting of erythrocytosis, suggesting primary erythrocytosis	4.0–27.0 mIU/mL
July 2025	*CALR *mutation	Negative	No evidence of *CALR*-associated myeloproliferative neoplasm	-
July 2025	*MPL* mutation	Negative	No evidence of *MPL*-associated myeloproliferative neoplasm	-
July 2025	Myeloid NGS panel	No pathogenic variants detected	No clonal mutation detected despite persistent erythrocytosis	-
July 2025	LDH	305 (elevated)	Elevated LDH suggesting increased cellular turnover	120–250 U/L
July 2025	Ferritin	476	Elevated ferritin consistent with prior iron repletion	15–150 ng/mL
July 2025	Intrinsic factor antibody	2.2 (positive)	Supports diagnosis of pernicious anemia	AU
July 2025	Anti-parietal cell antibody	38.4 (positive)	Supports autoimmune gastritis/pernicious anemia	U/mL
December 2023	Vitamin D	39	Within normal range	(ng/mL)
Early October 2023	Folate	15.2	Normal folate level	>4 ng/mL
Mid-October 2023	*JAK2* mutation	Negative	No evidence of *JAK2*-associated myeloproliferative neoplasm	-
Early October 2020	Vitamin B12	230	Borderline low vitamin B12 level	200–900 pg/mL

Given the discordance between the clinical phenotype (persistent erythrocytosis with suppressed erythropoietin) and the absence of molecular markers, bone marrow biopsy was pursued. An initial bone marrow biopsy performed in January 2025 demonstrated hypercellular marrow with trilineage hematopoiesis and megakaryocytic hyperplasia with atypia, initially interpreted as reactive (Table 6). However, due to persistent erythrocytosis and continued clinical suspicion for a myeloproliferative neoplasm, a repeat bone marrow biopsy was performed in March 2026.

**Table 6:** Bone marrow histopathologic findings in January 2025 The peripheral blood values shown in this table were extracted directly from the pathology report corresponding to the bone marrow biopsy performed on January 15, 2025. Hb, hemoglobin

Category	Findings	Clinical interpretation
Final diagnosis	No evidence of hematolymphoid neoplasm	No definitive leukemia, lymphoma, or myeloproliferative neoplasm identified
Peripheral blood findings	Slight polycythemia (Hb 16.3 g/dL); mild neutrophilia (6,500/µL); monocytosis (700/µL)	Consistent with erythrocytosis and mild myeloid expansion
Bone marrow cellularity	Hypercellular marrow	Increased hematopoietic activity
Lineage proliferation	Trilineage panhyperplasia	Expansion of erythroid, myeloid, and megakaryocytic cell lines
Megakaryocyte morphology	Megakaryocytic hyperplasia with morphologic atypia	Findings that may be seen in myeloproliferative neoplasms
Flow cytometry	No immunophenotypic evidence of hematolymphoid neoplasm; increased hematogones	No abnormal lymphoid or myeloid population detected
Cytogenetics	Normal female karyotype: 46,XX	No chromosomal abnormalities detected
Molecular testing	Negative for *JAK2* V617F, *JAK2 *exon 12–13, *CALR*, and *MPL *mutations	No driver mutations detected
Overall interpretation	Marrow changes interpreted as reactive in the absence of clonal markers	Findings may reflect secondary erythrocytosis rather than a primary myeloproliferative disorder
Pathologist recommendation	Repeat bone marrow biopsy if clinical suspicion persists	Further evaluation recommended if erythrocytosis remains unexplained

The repeat biopsy demonstrated marked hypercellularity with trilineage panmyelosis, prominent erythroid proliferation, and clusters of enlarged pleomorphic megakaryocytes with hyperlobulated nuclei: morphologic findings characteristic of PV, thereby establishing the diagnosis despite the absence of driver mutations [13]. Figure 1 serves as a visual representation of the bone marrow morphology and immunohistochemical findings, demonstrating a hypercellular marrow with iron staining, the presence of CD34-positive blasts, and lineage-specific markers highlighting granulocytic (CD15), erythroid (CD71), and megakaryocytic (CD61) populations, taken from the bone marrow morphology report. Additional imaging revealed mild splenomegaly on abdominal ultrasound (13.5 cm), consistent with myeloproliferative disease.

**Figure 1:**
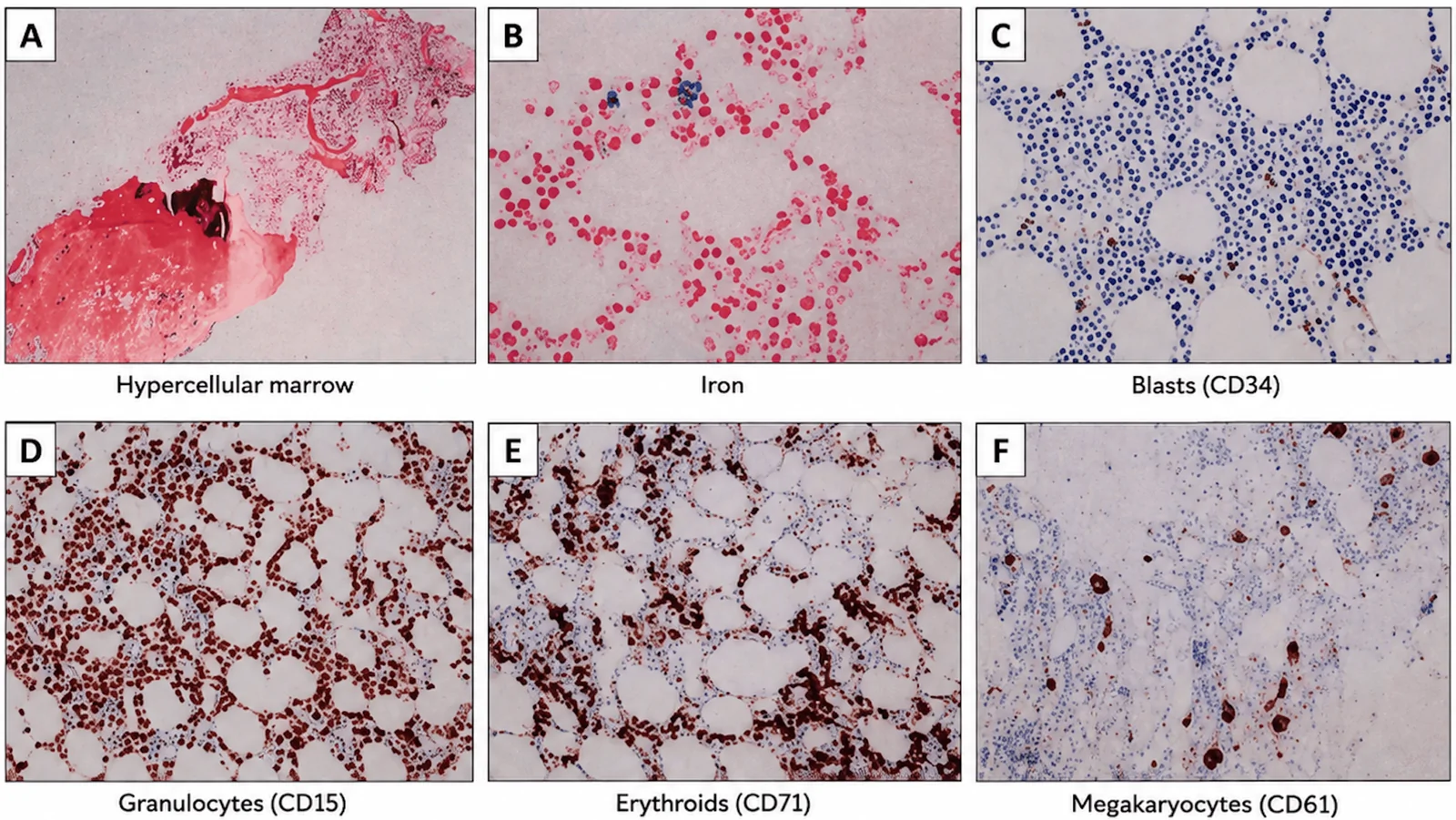
Bone marrow morphology and immunohistochemical findings (A) Hypercellular bone marrow demonstrating marked trilineage hematopoiesis (hematoxylin and eosin stain). (B) Iron stain showing preserved iron stores without ring sideroblasts. (C) CD34 immunostain demonstrating scattered blasts without increased blast population. (D) CD15 immunostain highlighting granulocytic lineage proliferation. (E) CD71 immunostain demonstrating prominent erythroid proliferation. (F) CD61 immunostain highlighting enlarged pleomorphic megakaryocytes with hyperlobulated nuclei.

The clinical course can be divided into three distinct phases: (1) initial severe iron deficiency anemia and pernicious anemia with hemoglobin levels of 5.2-6.1 g/dL requiring transfusion, (2) hematologic correction following iron and vitamin B12 repletion with normalization of hemoglobin, and (3) subsequent development of progressive erythrocytosis with hemoglobin rising to 19.8 g/dL and hematocrit to 58.7% (Table 7).

**Table 7:** Clinical timeline of disease progression Hb, hemoglobin

Date / period	Clinical event	Key findings	Clinical interpretation
May 2020	Initial presentation with severe anemia	Hb 5.2–6.1 g/dL; iron deficiency labs	Severe iron deficiency anemia
May to June 2020	Initiation of IV iron therapy	Low iron, low ferritin, high TIBC	Treatment of iron deficiency
October 2020	Diagnosis of B12 deficiency	Low B12; positive intrinsic factor antibodies	Pernicious anemia diagnosed
2021–2022	Maintenance therapy (iron + B12)	Normalized hemoglobin; symptom resolution	Hematologic recovery phase
2023	Recurrence of B12 deficiency	Persistent malabsorption	Ongoing pernicious anemia
2023–2024	Development of erythrocytosis	Hb rising to 17–19 g/dL	Emerging primary erythrocytosis
January 2025	First bone marrow biopsy	Hypercellular marrow; trilineage hematopoiesis	Initially interpreted as reactive
2025–2026	Persistent erythrocytosis	Low EPO; negative *JAK2*/*CALR*/*MPL*	Suspicion for myeloproliferative neoplasm
March 2026	Repeat bone marrow biopsy	Panmyelosis; megakaryocytic atypia	Findings consistent with polycythemia vera
2026 (current)	Ongoing follow-up	Hb ~18–19 g/dL; stable	Triple-negative polycythemia vera

Overall, the constellation of suppressed erythropoietin, persistent erythrocytosis, characteristic bone marrow morphology, and exclusion of secondary causes supports a diagnosis of *JAK2*-unmutated PV according to WHO criteria (major criteria 1 and 2 plus minor criterion) [1,12]. The marked increase in hemoglobin from 5.2 g/dL to 19.8 g/dL following nutritional repletion exceeded what would be expected from correction of deficiencies alone and was the key clinical clue prompting evaluation for an underlying myeloproliferative disorder [5]. The patient is classified as high-risk based on age greater than 60 years [14]. She remains clinically stable on continued vitamin B12 supplementation for pernicious anemia and aspirin 81 mg daily. Given her current hematocrit of 58.7%, which exceeds the evidence-based target of less than 45%, therapeutic phlebotomy is indicated to reduce thrombotic risk [15]. Cytoreductive therapy with hydroxyurea or pegylated interferon-alpha should also be considered given her high-risk status [1,14].

## Discussion

This case illustrates a diagnostically challenging presentation of PV that remained masked for several years due to the coexistence of iron deficiency anemia and pernicious anemia secondary to autoimmune gastritis. The patient initially presented with severe chronic anemia requiring multiple transfusions, and only after correction of both iron and vitamin B12 deficiencies did persistent erythrocytosis become apparent. Further complicating the diagnosis, molecular testing for common myeloproliferative driver mutations (*JAK2*, *CALR*, and *MPL*) was negative. Ultimately, the diagnosis relied on clinical findings, suppressed erythropoietin levels, and characteristic bone marrow morphology demonstrating hypercellularity with trilineage panmyelosis and megakaryocytic atypia. This case highlights a rare convergence of dual masking mechanisms, in which both iron deficiency and ineffective erythropoiesis from vitamin B12 deficiency obscured the underlying myeloproliferative process.

Pernicious anemia results from autoimmune destruction of gastric parietal cells, leading to loss of intrinsic factor production and impaired vitamin B12 absorption, which causes megaloblastic anemia along with achlorhydria and hypergastrinemia [8]. The gastritis arises from the activation of pathologic Th1 CD4 T cells directed against gastric H+/K+ ATPase, the proton pump normally resident on gastric mucosal secretory membranes [8,16]. Loss of gastric acid also impairs iron absorption, as gastric acid is required to reduce dietary ferric iron to the absorbable ferrous form [16]. In autoimmune gastritis, iron deficiency typically develops first and may precede vitamin B12 deficiency by years to decades, which may explain the progression from microcytic to macrocytic anemia observed in our patient [8].

Iron deficiency commonly accompanies PV and may obscure erythrocytosis by reducing hemoglobin concentration and hematocrit [3]. The resulting microcytosis lowers the hematocrit despite an elevated red blood cell count [3]. In addition, PV frequently causes plasma volume expansion, which further masks the true erythrocytosis by diluting the red cell concentration; in many patients, especially women, the hematocrit appears to be normal despite a significantly elevated red cell mass [4]. Together, these mechanisms produce "masked PV," in which hemoglobin and hematocrit appear normal or only mildly elevated despite true PV [4]. Once iron stores are replenished, the underlying erythrocytosis becomes clinically apparent as hemoglobin and hematocrit levels increase [3]. Masked PV can lead to significant diagnostic delays, during which patients remain at risk for thrombotic complications including stroke, myocardial infarction, and venous thromboembolism [14].

Most patients with PV harbor mutations in *JAK2*, with approximately 96% carrying the *JAK2* V617F mutation in exon 14 and an additional 3% having mutations in exon 12 [1]. However, a small subset of patients (approximately 2%) lacks mutations in *JAK2*, *CALR*, and *MPL* [1]. In these cases, diagnosis relies primarily on clinical findings, laboratory parameters (hemoglobin greater than 16.5 g/dL in men or greater than 16.0 g/dL in women, and low erythropoietin), and bone marrow morphology [1,12]. In this patient, the suppressed erythropoietin of 2.1 mIU/mL strongly supported a diagnosis of primary erythrocytosis rather than a secondary process, as secondary erythrocytosis is typically associated with elevated erythropoietin levels [1].

Prior to establishing the diagnosis of primary PV, a comprehensive evaluation to exclude secondary causes of erythrocytosis was performed. Secondary causes associated with elevated erythropoietin levels were systematically excluded: obstructive sleep apnea was ruled out by polysomnography, erythropoietin-secreting tumors such as renal cell carcinoma and hemangioblastoma were excluded by renal and abdominal imaging, and hypoxia-driven erythrocytosis was not identified [1]. In *JAK2*-unmutated cases, hereditary erythrocytosis must also be considered, including *EPOR* mutations (which present with low erythropoietin similar to PV), *VHL* mutations, *EGLN1* (PHD2) mutations, and *EPAS1* (HIF2A) mutations [17]. The patient's late onset of erythrocytosis (unmasked only after nutritional repletion at age 68), absence of family history of erythrocytosis, and characteristic bone marrow morphology argued against a hereditary etiology, as hereditary erythrocytosis typically presents as lifelong erythrocytosis [18]. However, expanded next-generation sequencing panels for hereditary erythrocytosis may be considered in cases with high clinical suspicion and negative standard workup [17].

Given the discordance between the clinical phenotype (persistent erythrocytosis with suppressed erythropoietin) and the absence of molecular markers, bone marrow biopsy was pursued. An initial bone marrow biopsy performed in January 2025 demonstrated hypercellular marrow with trilineage hematopoiesis and megakaryocytic hyperplasia with atypia but lacked the characteristic clustering of pleomorphic megakaryocytes, leading to its initial interpretation as reactive in the setting of negative molecular testing. However, due to persistent erythrocytosis and continued clinical suspicion for a myeloproliferative neoplasm, a repeat bone marrow biopsy was performed.

The repeat biopsy demonstrated marked hypercellularity with trilineage panmyelosis, prominent erythroid proliferation, and clusters of enlarged pleomorphic megakaryocytes with hyperlobulated nuclei, morphologic findings that represent a major diagnostic criterion for PV and help distinguish it from secondary erythrocytosis [13]. Additional imaging revealed mild splenomegaly (13.5 cm), consistent with myeloproliferative disease.

This case is notable for several reasons. First, the dual masking mechanism of iron deficiency combined with pernicious anemia simultaneously concealing PV has not been previously reported in the literature. Second, the dramatic hemoglobin swing from 5.2 g/dL to 19.8 g/dL (a change of 14.6 g/dL) following nutritional repletion far exceeded what would be expected from correction of deficiencies alone and served as the key clinical clue prompting evaluation for an underlying myeloproliferative disorder. Third, the case demonstrates the importance of repeat bone marrow biopsy when clinical suspicion persists despite initial findings interpreted as reactive.

Regarding management, the CYTO-PV trial demonstrated that maintaining hematocrit below 45% significantly reduced cardiovascular death and major thrombosis [15]. Given this patient's current hematocrit of 58.7%, therapeutic phlebotomy is indicated. Additionally, as a high-risk patient (age greater than 60 years), cytoreductive therapy should be considered, with hydroxyurea and pegylated interferon-alpha as first-line options [1,14]. The patient's continued requirement for vitamin B12 supplementation for pernicious anemia adds complexity to management, as interruption of B12 therapy could theoretically suppress erythropoiesis and mask the true extent of the myeloproliferative process.

## Conclusions

This case demonstrates the diagnostic complexity of masked PV in the setting of combined nutritional deficiencies secondary to autoimmune gastritis. The evolution from severe transfusion-dependent anemia (hemoglobin 5.2 g/dL) to marked erythrocytosis (hemoglobin 19.8 g/dL) following correction of iron deficiency and pernicious anemia illustrates how coexisting conditions can obscure an underlying myeloproliferative disorder. To the authors’ knowledge, this dual masking mechanism has not been previously reported. Several teaching points emerge from this case. Erythrocytosis following correction of nutritional deficiencies should prompt evaluation for an underlying myeloproliferative neoplasm. In *JAK2*-unmutated cases, bone marrow morphology and suppressed erythropoietin are particularly important, and repeat biopsy may be necessary when clinical suspicion persists despite initially reactive findings. Autoimmune gastritis should also be recognized as a potential cause of concurrent iron and vitamin B12 deficiency capable of masking underlying hematologic disease. Management should follow established PV risk-stratification principles, including maintaining hematocrit below 45% and considering cytoreductive therapy in high-risk patients. Clinical-pathologic correlation remains essential when the clinical picture suggests a myeloproliferative process despite negative initial workup.
